# The effectiveness of Zhi-Shi plaster acupoint therapy combined with lactulose in the treatment of geriatrics functional constipation: a randomized controlled trial

**DOI:** 10.3389/fneur.2025.1580163

**Published:** 2025-12-04

**Authors:** Ying Wang, XiXia Zhang, XueFei Ding, Hui Li, JunQuan Xia, MengYan Zhou, YongZhi Hua, KeXuan Wu, LongShu Zhang, LiRong Zhou

**Affiliations:** 1 School of Nursing, Nanjing University of Chinese Medicine, Nanjing, China; 2 Affiliated Hospital of Integrated Traditional Chinese and Western Medicine, Nanjing University of Chinese Medicine, Jiangsu, China

**Keywords:** acupoint therapy, functional constipation, older adult, lactulose, Zhi-Shi plaster

## Abstract

**Objective:**

This study aimed to evaluate the effectiveness and adverse effects of Zhi-Shi plaster acupoint therapy combined with Lactulose in the Treatment of geriatrics Functional Constipation.

**Methods:**

A total of 62 geriatrics Functional Constipation patients were randomly assigned to the intervention group (*n* = 31) and control group (*n* = 31). The intervention group received oral lactulose (20 mL daily for 14 consecutive days) plus Zhi-Shi plaster acupoint therapy (for 4 h daily at the following acupoints: CV8, bilateral ST25, and bilateral SP15). The control group received oral lactulose plus placebo acupoint therapy during the same period. The primary outcomes included the clinical efficacy and the Constipation Symptom Scoring Scale (CSS). Secondary outcomes included the weekly count of complete spontaneous bowel movements (CSBMs), the Bristol Stool Form Scale (BSFS), the Patient Assessment of Constipation-Symptoms (PAC-SYM), the Constipation Quality of Life Scale (PAC-QOL), and adverse effects. Assessments were conducted at baseline, the week1, the week2, and the week6, with data analysis using generalized estimating equations. And the outcome assessors and statistician was blinded.

**Results:**

After treatment, the overall clinical efficacy was higher in the intervention group than in controls (96.77% vs. 83.87%, *p* = 0.03). The intervention group demonstrated superior improvements across outcomes compared to the control group: constipation symptoms (CSS: coefficient = −3.36, 95% CI: −4.68 to −2.03, *p* < 0.001), frequency of complete spontaneous bowel movements (CSBMs: coefficient = 0.59, 95% CI: 0.40 to 0.79, *p* < 0.001), and stool consistency (BSFS: coefficient = 0.45, 95% CI: 0.20 to 0.70, *p* < 0.001). Similarly, significant reductions in patient-assessed symptom severity (PAC-SYM: coefficient = −0.72, 95% CI: −0.91 to −0.52, *p* < 0.001) and greater enhancements in quality of life (PAC-QOL: coefficient = −0.34, 95% CI: −0.43 to −0.24, *p* < 0.001) were observed. Finally, the incidence of adverse reactions was lower in the intervention group (3.22% vs. 19.35%, *p* = 0.015).

**Conclusion:**

The combination of lactulose and Zhi-Shi plaster acupoint therapy for the treatment of geriatrics Functional constipation can significantly improve clinical efficacy, reduce adverse effects, alleviate symptoms and enhance quality of life.

**Clinical trial registry:**

This trial was registered in the International Traditional Medicine Clinical Trial Registry (ITMCTR; registration number: ITMCTR2024000401).

## 1 Introduction

Functional constipation (FC) is a gastrointestinal disorder characterized by difficulty in defecation, reduced bowel movement frequency, and a sensation of incomplete evacuation (1). Its diagnosis is based on Rome IV criteria, complemented by patient-reported symptoms (2). The global prevalence of FC among individuals aged ≥60 years is estimated to be 22.0% (3), with even higher rates observed in those aged ≥65 years (16% in men and 26% in women). FC might lead to severe anorectal complications (4, 5), and the quality of life for older adult individuals is often impaired, placing a heavy economic burden on both patients and healthcare systems (6–8). Consequently, finding effective preventive and therapeutic measures has become an important topic in clinical research.

Lactulose, as an osmotic laxative, is effective in treating older adult FC (9). However it may lead to discomfort, such as nausea and bloating (10). Experimental findings indicate that the incidence of adverse reactions of lactulose, including bloating, nausea and vomiting reached as high as 16.92% (11). Although lactulose can temporarily improve intestinal motility, its long-term efficacy is limited, with a relapse rate of 22.2% within 1 month of discontinuation (12–14). The pronounced adverse effects, coupled with the frequent recurrence after discontinuation, restrict the clinical use of lactulose (2).

In response to the limitations of monotherapy, a synergistic strategy integrating lactulose with TCM acupoint plasters has been advanced (12, 15). This approach is grounded in the established efficacy of a wide spectrum of non-pharmacological methods for constipation, which include physical exercise, abdominal massage, TENS, acupuncture, posture education, and various TCM nursing techniques (16, 17). It specifically capitalizes on the key advantages of acupoint plasters: their non-invasive nature, suitability for self-care in the older adult, and sustained transdermal mode of action (18, 19), with the goal of achieving superior enhancement of abdominal motility and reduction in recurrence rates.

However, many conventional TCM plasters for FC rely on anthraquinone-based constituents (e.g., rhubarb), which are often associated with mucosal irritation, allergic reactions, and poor tolerance among older adult populations (20–22), which limits their clinical utility and safety profile. This limitation underscores the need to develop novel TCM plaster formulations that are not only effective but also specifically designed for safety and tolerability in geriatric FC patients.

According to TCM theory, FC in the older adult arises from Qi stagnation and intestinal dryness, necessitating treatments that regulate Qi, relieve stagnation, and moisten the intestines (23). The Zhi-Shi plaster is a novel formulation designed based on this principle. It incorporates: Zhi Shi (Aurantii Fructus Immaturus), which contains flavonoids, alkaloids, and essential oils with Qi-regulating effects (24, 25); Bing Lang (Arecae Semen), which enhances gastrointestinal motility (26); Ku Xing Ren (Armeniacae Semen Amarum), Tao Ren (Persicae Semen), and Gua Lou Zi (Trichosanthis Semen), which are rich in fatty oils for intestinal lubrication (27–29); and Dang Gui (Angelicae Sinensis Radix), which nourishes blood and promotes bowel movement (30). Thus, these components act synergistically to address gastrointestinal dysmotility and bloating while potentially reducing lactulose-induced adverse effects.

Beyond composition, the plaster utilizes acupoint application at Shenque (CV8), Tianshu (ST25), and Daheng (SP15), leveraging meridian conduction and transdermal absorption to enhance local drug concentration and gastrointestinal motility. Although preliminary evidence indicates that Zhi-Shi plaster significantly reduces in colon cleansing time from 301.41 min to 247.31 min, indicating more efficient colonic clearance (31), its efficacy and safety in older adult FC patients—particularly in combination with lactulose—remain scientifically unvalidated.

This randomized controlled trial aims to evaluate the effects of combining Zhi-Shi plaster acupoint therapy with lactulose, compared to lactulose alone, on bowel function, quality of life, and adverse effects in geriatric patients with FC, in order to provide high-quality clinical evidence.

## 2 Materials

### 2.1 Trial design

This was a prospective, single-center, randomized, placebo-controlled clinical trial conducted from November 2023 to June 2024 at the Affiliated Hospital of Integrated Traditional Chinese and Western Medicine, Nanjing University of Chinese Medicine. The study protocol was approved by the hospital’s Medical Ethics Committee (Approval Code: 2023-LWKYZ-078) and was also registered with the International Traditional Medicine Clinical Trial Registration Platform (registration code: ITMCTR2024000401). All participants were given written consent informs. The study adhered to clinical practice guidelines and the ethical principles outlined in the Declaration of Helsinki.

### 2.2 Participants

We enrolled inpatient and outpatient participants from the Department of Gastroenterology of the Affiliated Hospital of Integrated Traditional Chinese and Western Medicine, Nanjing University of Chinese Medicine.

#### 2.2.1 Inclusion criteria

(1) Aged ≥60 years; (2) fulfillment of the diagnostic criteria for FC (Rome IV); (3) ability to record their signs and symptoms themselves or with the help of their families.

#### 2.2.2 Exclusion criteria

(1) The presence of organic diseases related to constipation, cardiovascular, neurological, hematologic or metabolic diseases, mental disorders, malignant tumor, history of alcohol use, or substance abuse; (2) Abdominal skin damage or infection.

### 2.3 Interventions

Both groups received the treatment of lactulose (manufactured by Nanjing Hengsheng Pharmaceutical Co., Ltd., Approval no. H20243233, Specification: 100 mL: 66.7 g), taken once with breakfast, the dose was 20 mL/d for 2 weeks.

#### 2.3.1 Intervention group

**Figure 1:**
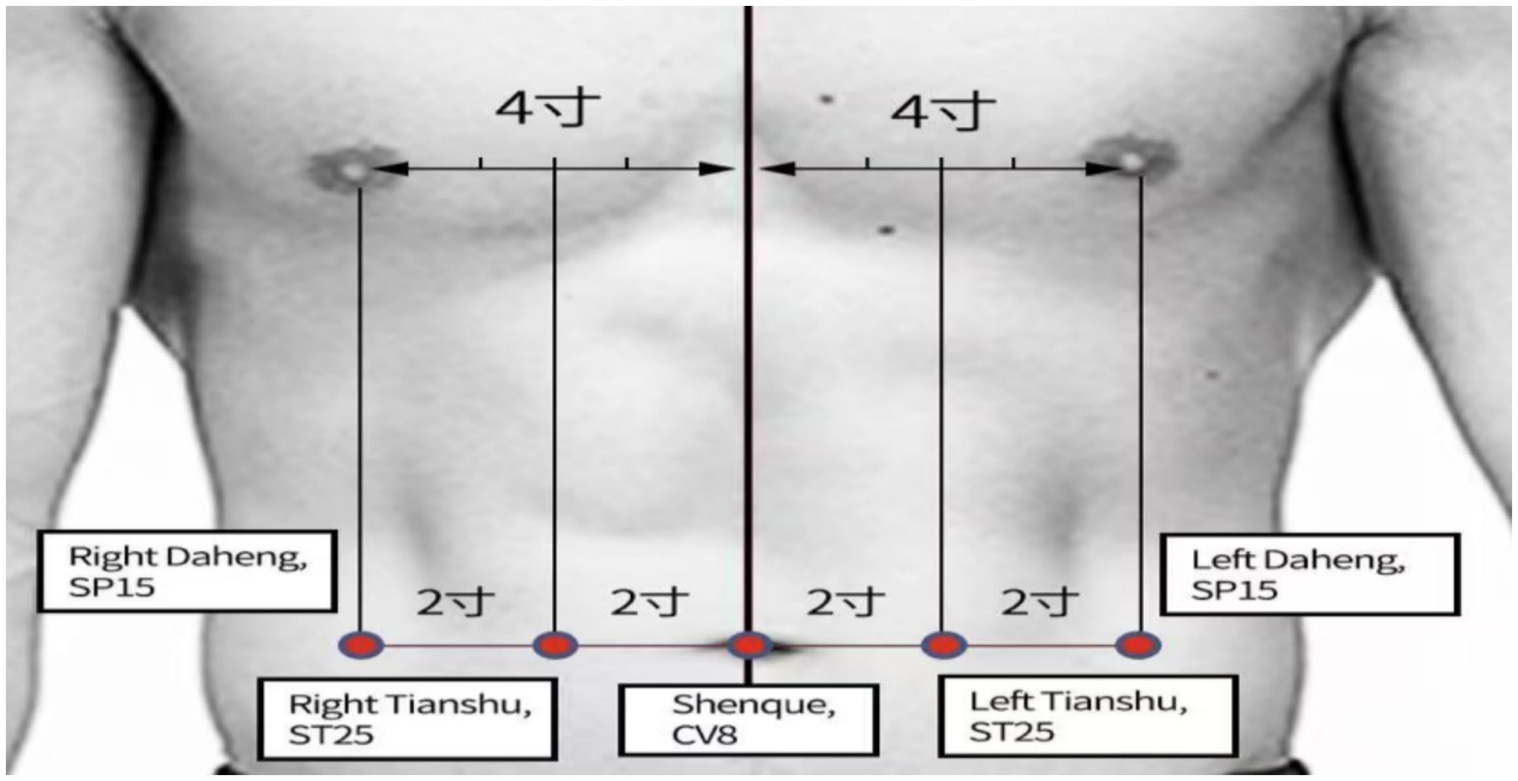
Acupoint selection for relieving constipation.

Patients allocated to the intervention group were treated with Lactulose and Zhi-Shi plaster acupoint therapy. The Zhi-Shi plaster was prepared with powders made from Zhishi (*Aurantii Fructus immaturus*), betel nut (*Arecae Semen*), bitter apricot (*Pruni Armeniacae Semen*), peaches (*Persicae Semen*), Angelica (*Angelicae Sinensis Radix*), and trichosanthes seed (*Trichosanthis Fructus*) mixed with petroleum jelly. A 10 g portion of Zhi-Shi plaster was attached to patient’s CV8, bilateral ST25, and bilateral SP15 (Figure 1; Table 1) by using acupoint patches, with 4 h daily for 2 weeks.

**Table 1:** Rationale for acupoint selection in the treatment of FC.

Acupoint	Anatomical location	Rationale for selection in this study
CV8 (Shenque)	In the center of the umbilicus.	Selected: It has distinct physiological features and rich vascular and neural networks, transdermal drug delivery at Shenque results in a higher absorption rate and faster drug permeation (46), which is particularly suitable for older adult patients.
ST25 (Tianshu)	On the abdomen, 2 cun lateral to the center of the umbilicus.	Selected: As the Front-Mu point of the large intestine, it is a pivotal point for regulating intestinal Qi and treating constipation (47, 48). Its location directly over the colon makes it ideal for transdermal drug delivery (49).
SP15 (Daheng)	On the abdomen, 4 cun lateral to the center of the umbilicus.	Selected. A key point for regulating the spleen and resolving stagnation. It synergizes with ST25 to promote bowel movements, forming a core abdominal point combination.

##### 2.3.1.1 Application time of the patch

Propagated contractions of the colon are classified as low (5–40 mmHg) or high amplitude (>75 mmHg). These contractions occur in the cecum or ascending colon upon morning awakening, resulting in the mass movement of colonic contents. In TCM theory, the 12 earthly branches are matched to the Zang-Fu organs and meridian collaterals. The order of Qi and blood circulation through the meridians corresponds to the 12 traditional Chinese double-hour periods. For instance, Qi and blood are most abundant in the large intestine meridian during the Maoshi period (5:00–7:00 a.m.). Thus, optimum effects can be achieved if acupoint therapy is executed during the corresponding periods. According to clinical technical guidelines for acupoints therapy, the typical application time is 4–6 h (32). The study also found that a 4-h application duration for older adult patients with FC resulted in clear therapeutic effects and a low incidence of adverse reactions (33). Therefore, the time of Zhi-Shi plaster acupoint therapy was set at 5:00–7:00 a.m., with the application time for 4 h daily.

#### 2.3.2 Control group

Patients received lactulose and placebo acupoint therapy. The placebo was prepared with flour pill, food coloring and petroleum jelly to make it seems like Zhi-Shi plaster. The placebo was also applied to the patient’s CV8, bilateral ST25, and bilateral SP15. Similar to the Zhi-Shi plaster acupoint therapy, placebo acupoint therapy was performed at 5–7 a.m. for 2 consecutive weeks, each lasting 4 h.

### 2.4 Assessments and outcomes

#### 2.4.1 The primary outcome measures

##### 2.4.1.1 The clinical efficacy

According to the “Expert consensus on the traditional Chinese medicine diagnosis and treatment of constipation (2024)” the clinical efficacy is classified into four levels:

1. Cured: the CSS scores decreased by ≥95%;
2. Markedly effective: the CSS scores decreased by ≥70%;
3. Effective: the CSS scores decreased by ≥30%;
4. Ineffective: the CSS scores decreased by <30%.

##### 2.4.1.2 Constipation Symptom Scoring Scale (CSS)

The Constipation Symptom Scoring Scale (CSS) consists of five items that assess the following constipation symptoms: stool consistency, bowel movement frequency, time spent on defecation, difficulty and excessive straining during defecation, and abdominal bloating. Each item is assigned a score of 0 (never), 2 (occasionally), 3 (frequent), and 4 (persistent). The total score range from 0 to 15 points, with higher scores indicating greater severity of constipation symptoms.

#### 2.4.2 Secondary outcome measures

##### 2.4.2.1 CSBMs

The weekly frequency of complete spontaneous bowel movements (CSBMs) was evaluated. A weekly CSBM count of ≥3 was defined as indicative of normal bowel function (34).

##### 2.4.2.2 BSFS

The Bristol Stool Form Scale (BSFS) (35) categorizes human stool types from hardest (type 1) to softest (type 7) as an ordinal scale. When paired with additional constipation-related symptoms, types 1 and 2 are deemed to be abnormally hard feces, while types 6 and 7 are deemed to be abnormally loose/liquid stools (in conjunction with other symptoms indicative of diarrhea). As a result, types 3, 4, and 5 are typically considered to be the “regular” stool types (36). The BSFS demonstrated excellent interclass reliability, with interclass correlation coefficients of 0.88 (95% CI: 0.86–0.90, *p* < 0.001) and 0.89 (95% CI: 0.86–0.91, *p* < 0.001) (35). The scale also demonstrated good internal consistency, with a Cronbach’s alpha of 0.88 (37).

##### 2.4.2.3 Pac-SYM

The PAC-SYM scale (38) comprises 12 items, divided into three subscales of abdominal symptoms (four items), rectal symptoms (three items), and stool symptoms (five items). On a scale from 0 to 4, with 4 denoting the most severe symptoms. The total score range from 0 to 48 points, with higher scores indicating more severe constipation. The PAC-SYM showed strong test–retest reliability (r = 0.86) and good internal consistency (Cronbach’s *α* = 0.91).

##### 2.4.2.4 Pac-QOL

The PAC-QOL scale (39) acknowledges constipation-related worries and concerns (11 items), physical discomfort (four items), psychosocial discomfort (eight items), and satisfaction (five items), with the answer scale for each item was recoded to 0–4. The PAC-QOL showed high test–retest reliability (*r* = 0.84) and good internal consistency (Cronbach’s *α* = 0.93).

##### 2.4.2.5 Adverse effects

Adverse effects includes nausea, abdominal bloating and diarrhea (≥3 bowel movements per day with watery stools) (11–14), were evaluated during the study using a patient-reported Adverse Effect Questionnaire.

### 2.5 Assessment time points

The above indices were evaluated at baseline, during the treatment at weeks 1 and 2, and at the end of the 4-week follow-up period (week 6).

### 2.6 Sample size

The sample size was determined using the two-sided t-test to assess the difference between the mean of the two groups in G*Power 3.1.9.2, with an effect size of 0.8 (40), *α* of 0.05, and power of 0.8, and the confidence level of 95%. The sample size was set at 62 (31 patients each in the intervention and control groups) to allow for an attrition rate of 20%.

### 2.7 Randomization and blinding

Eligible participants were randomly assigned to the intervention or control group in a 1:1 ratio. The randomization sequence was generated by an independent statistician using SPSS Statistics version 26.0 with randomly permuted block sizes of 3 and 5. The sequence was concealed using sequentially numbered, opaque, sealed envelopes (SNOSE), which were opened only after the participant had completed the baseline assessment and was formally enrolled in the trial.

The practitioners responsible for applying the plasters could not be blinded to group assignment due to the distinctive herbal aroma of the Zhi-Shi plaster. However, we mitigated the risk of bias in outcome assessment and data analysis by blinding the outcome assessors and statistician.

### 2.8 Statistical analysis

Data analysis was performed using SPSS (version 26.0). Continuous variables were expressed as mean and standard deviations, except for serious skewed variables. Serious skewed variables refer to mean/SD < 2 and stated as medians and upper and lower quartiles (41). Categorical variables were presented as [*n* (%)] and compared using the *χ*^2^-test (*p*-values<0.05). The outcome measures were analyzed using generalized estimating equations (GEE). Statistical significance was set at *p* < 0.05.

## 3 Results

### 3.1 Participant flow

**Figure 2:**
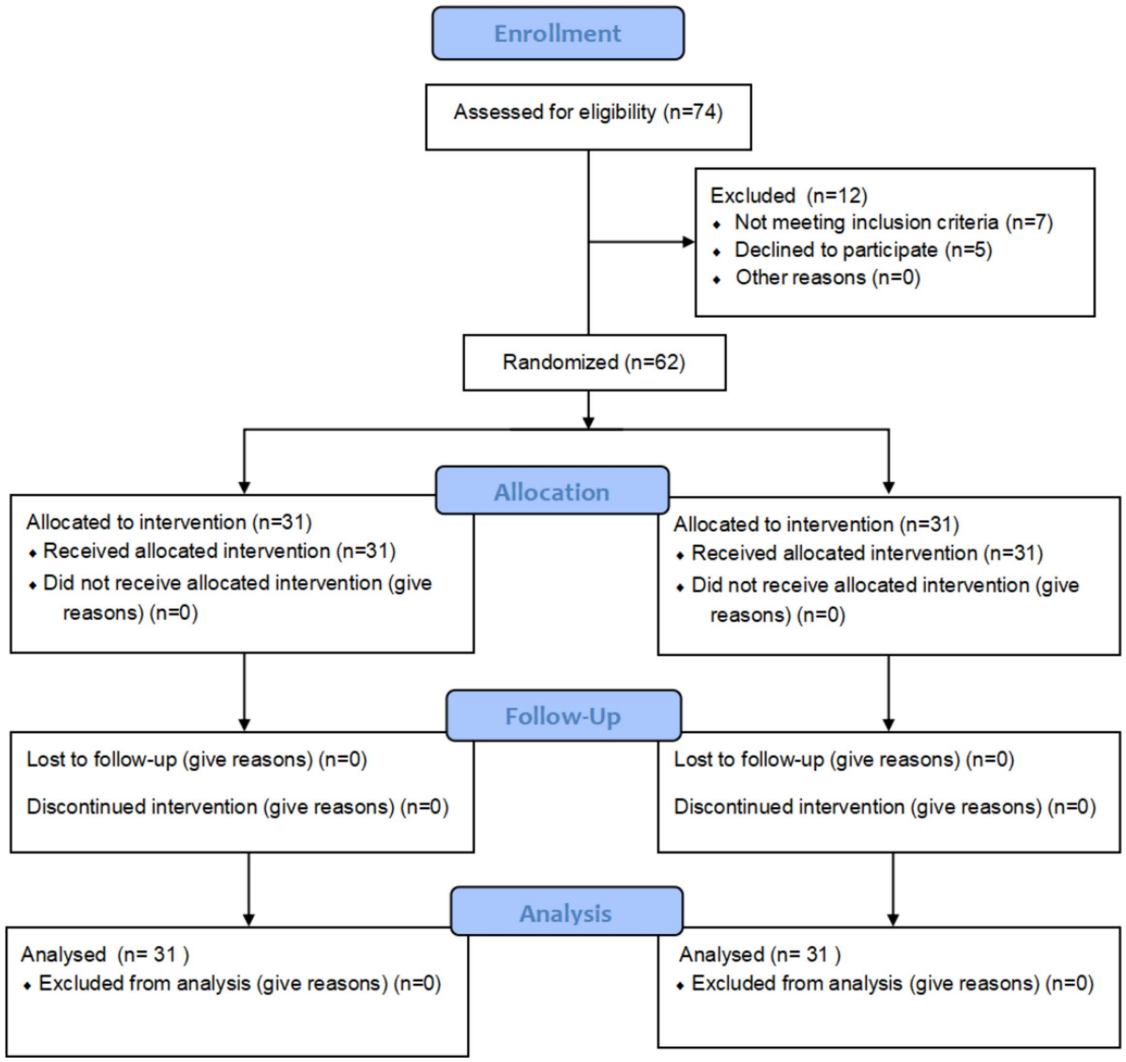
Study procedures.

Seventy-four older adult patients presenting with a complaint of constipation were screened for eligibility. Sixty-two of these patients met the eligibility criteria and were enrolled in the study. They were then randomly allocated to either the intervention group or the control group, with 31 participants in each group. No dropouts occurred during the observation period, and complete data from all 62 patients was included in the final analysis. The patient enrollment flow diagram is presented in Figure 2.

### 3.2 Comparison of baseline characteristics

The mean age was 70.94 ± 4.09 years in the intervention group and 70.48 ± 4.49 years in the control group. No statistically significant differences in baseline demographic or clinical characteristics were observed between the two groups (all *p* > 0.05). Detailed baseline characteristics are presented in Table 2.

**Table 2:** Baseline characteristics of study participants.

Characteristic	Intervention (*n* = 31)	Control (*n* = 31)	*p*
Sex, *n* (%)			0.319*
Male	7 (22.6%)	4 (12.9%)	
Female	24 (77.4%)	27 (87.1%)	
Age(y),mean ± SD	70.94 ± 4.09	70.48 ± 4.49	0.68#
Duration of disease (y), [M (P25, P75)]	4 (2,10)	4 (3,10)	0.541##
The Constipation Symptom Scoring Scale [M (P25, P75)]	13 (11,14)	13 (11,14)	0.606##
CSBMs [M (P25, P75)]	1.68 (1,2)	1.90 (1,2)	0.168##
BSFS [M (P25, P75)]	1.68 (1,2)	1.61 (1,2)	0.809##
PAC-SYM [M (P25, P75)]	19 (17,27)	23 (20,27)	0.132##
PAC-QOL, mean ± SD	87.23 ± 8.65	89.06 ± 8.99	0.415#

*χ^2^ test.

#Independent *t*-test.

##Mann–Whitney U-test.

CSS, Constipation Symptom Scale, CSBMs, complete spontaneous bowel movements, BSFS, Bristol Stool Form Scale, PAC-SYM, Patient Assessment of Constipation-Symptoms, PAC-QOL, Patient Assessment of Constipation Quality of Life, GEE, generalized estimating equations.

### 3.3 Primary outcome measures

#### 3.3.1 Clinical efficacy

Following treatment, the overall clinical efficacy rate was significantly higher in the intervention group than in the control group (96.77% vs. 83.87%; *p* = 0.03). Detailed results are presented in Table 3.

**Table 3:** Comparison of the clinical efficacy rate between the two groups.

Group	*n*	Clinical recovery	Positive effect	Effective	Ineffective	Total effective
Intervention group	31	5 (16.13)	11 (35.48)	14 (45.16)	1 (3.22)	30 (96.77%)
Control group	31	1 (3.22)	12 (38.71)	13 (41.94)	5 (16.13)	23 (83.87%)
*Z*						8.00
*p*						0.03

*Compared with the control group, *p* = 0.03 < 0.05.

#### 3.3.2 Comparison of CSS scores in both groups

**Figure 3:**
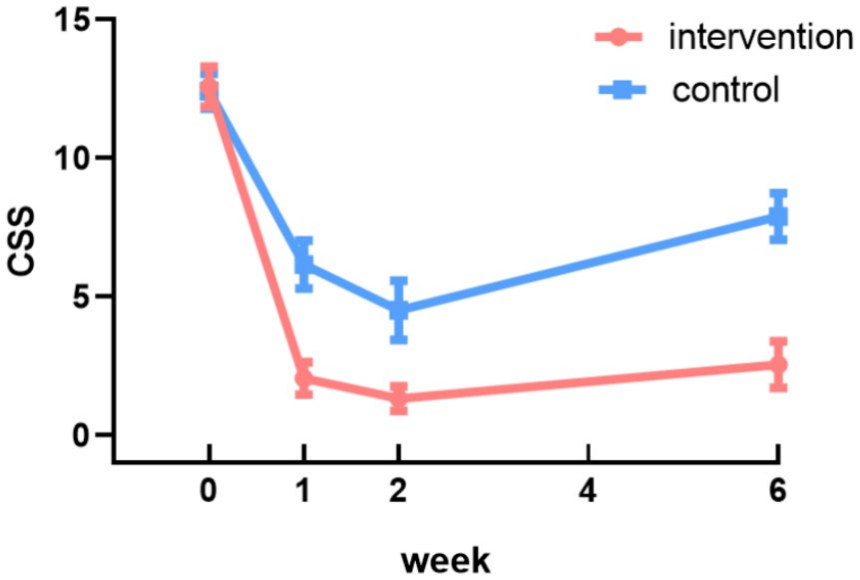
Changes in CSS scores in the intervention and control groups.

A GEE model was fitted using an exchangeable working correlation structure to account for within-subject correlations across repeated measurements, with robust standard errors applied for inference (42, 43). Using the baseline CSS scores of the control group as the reference, significant main effects of time and a significant group-by-time interaction were observed. No significant difference in CSS scores was detected between the two groups at baseline (*p* = 0.12). The significant interaction indicated that the pattern of change over time differed between groups. Specifically, while scores improved in both groups, the intervention group demonstrated a significantly greater reduction in CSS scores compared to the control group at all follow-up assessments (Week 1: coefficient = −4.26, 95% CI: −5.37 to −3.14; Week 2: coefficient = −3.36, 95% CI: −4.68 to −2.03; Week 6: coefficient = −5.52, 95% CI: −6.81 to −4.22; all *p* < 0.001) (Table 4). These results indicate that the improvement in constipation symptoms was significantly greater in magnitude in the intervention group compared to the control group (Figure 3).

**Table 4:** Comparison of CSS scores between the two groups.

Variable	CSS			p*
	B	SE	95% CI	
Group				
Control	Reference			
Intervention	0.16	0.46	(−0.74 to 1.07)	0.12
Time				
Baseline	Reference			
Week1	−6.26	0.44	(−7.13 to −5.39)	<0.001
Week2	−7.90	0.57	(−9.01 to −6.80)	<0.001
Week6	−4.52	0.46	(−5.42 to −3.61)	<0.001
Group × time				
Intervention × week1	−4.26	0.57	(−5.37 to −3.14)	<0.001
Intervention × week2	−3.36	0.68	(−4.68 to −2.03)	<0.001
Intervention × week6	−5.52	0.66	(−6.81 to −4.22)	<0.001

*Calculated using generalized estimating equations.

CSS, Constipation Symptom Scale; CSBMs, complete spontaneous bowel movements; BSFS, Bristol Stool Form Scale; PAC-SYM, Patient Assessment of Constipation-Symptoms; PAC-QOL, Patient Assessment of Constipation Quality of Life; GEE, generalized estimating equations; CI, confidence interval.

### 3.4 Secondary outcome measures

#### 3.4.1 Comparison of CSBMs, BSFS scores in both groups

**Figure 4:**
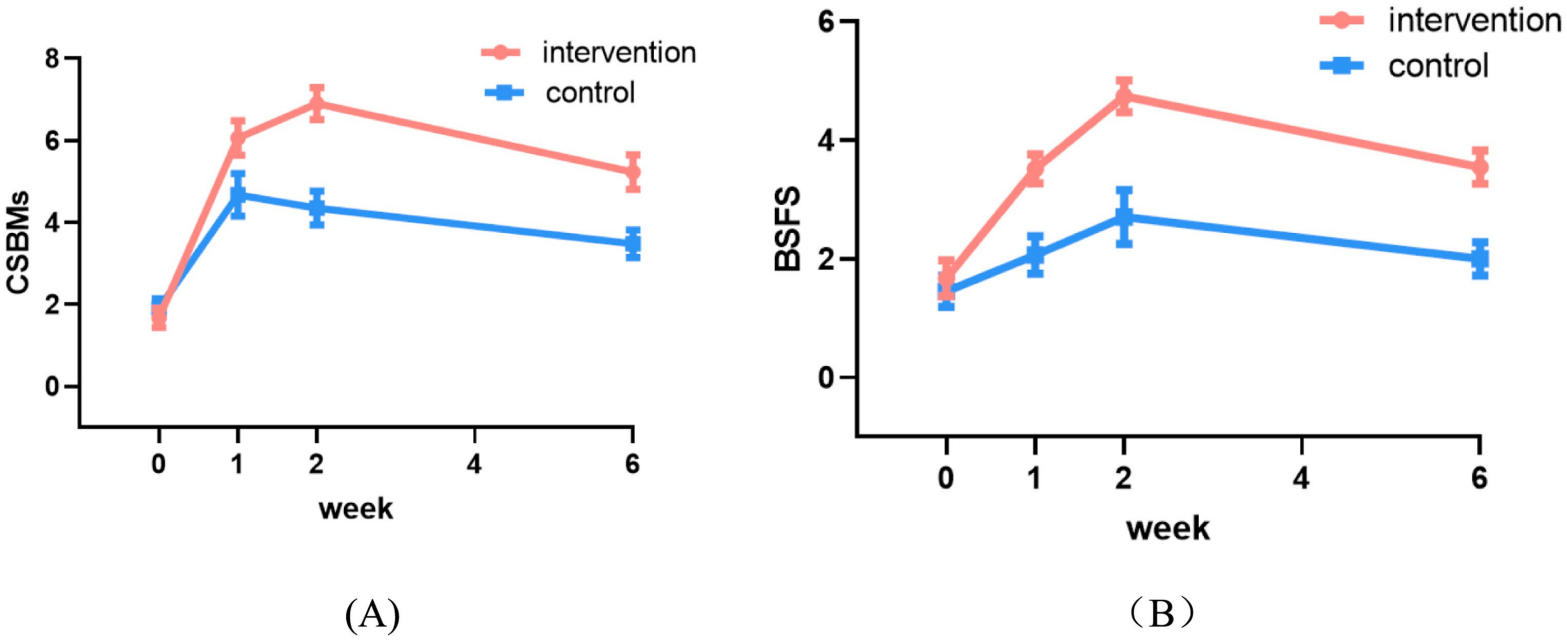
Changes in CSBMs **(A)** and BSFS **(B)** scores between the two groups.

Table 5 presents the comparison of CSBM and BSFS scores between the two groups. The significant interactions indicated that the patterns of improvement differed between the two groups. The intervention group demonstrated a significantly greater increase in CSBM frequency compared to the control group at all follow-up assessments (Week 1: coefficient = 0.39, 95% CI: 0.22 to 0.56; Week 2: coefficient = 0.59, 95% CI: 0.40 to 0.79; Week 6: coefficient = 0.53, 95% CI: 0.32 to 0.75; all *p* < 0.001) (Table 5). These results indicate that the improvement in spontaneous bowel movements was significantly greater in the intervention group (Figure 4A). Similarly, for stool consistency as measured by the BSFS, the intervention group showed a significantly greater improvement than the control group at all time points (Week 1: coefficient = 0.40, 95% CI: 0.19 to 0.62; Week 2: coefficient = 0.45, 95% CI: 0.20 to 0.70; Week 6: coefficient = 0.43, 95% CI: 0.20 to 0.67; all *p* < 0.001) (Table 5), indicating superior improvement in stool consistency (Figure 4B).

**Table 5:** Comparison of CSBMs and BSFS scores between the two groups.

Variable	CSBMs			p*	BSFS			p*
	B	SE	95%Cl		B	SE	95%Cl	
Group								
Control	Reference							
Intervention	−0.13	0.09	(−0.31 to 0.05)	0.17	0.15	0.12	(−0.10 to 0.39)	0.24
Time								
Baseline	Reference							
Week1	0.90	0.09	(0.73 to 1.07)	<0.001	0.34	0.07	(0.19 to 0.48)	<0.001
Week2	0.82	0.07	(0.68 to 0.96)	<0.001	0.59	0.10	(0.40 to 0.78)	<0.001
Week6	0.61	0.08	(0.45 to 0.76)	<0.001	0.32	0.08	(0.17 to 0.47)	<0.001
Group × time								
Intervention × week1	0.39	0.12	(0.12 to 0.56)	<0.001	0.40	0.11	(0.19 to 0.62)	<0.001
Intervention × week2	0.59	0.10	(0.40 to 0.79)	<0.001	0.45	0.13	(0.20 to 0.70)	<0.001
Intervention × week6	0.53	0.11	(0.32 to 0.75)	<0.001	0.43	0.12	(0.20 to 0.67)	<0.001

*Calculated using generalized estimating equations.

CSS, Constipation Symptom Scale; CSBMs, complete spontaneous bowel movements; BSFS, Bristol Stool Form Scale; PAC-SYM, Patient Assessment of Constipation-Symptoms; PAC-QOL, Patient Assessment of Constipation Quality of Life; GEE, generalized estimating equations; CI, confidence interval.

#### 3.4.2 Comparison of PAC-SYM, PAC-QOL scores in both groups

**Figure 5:**
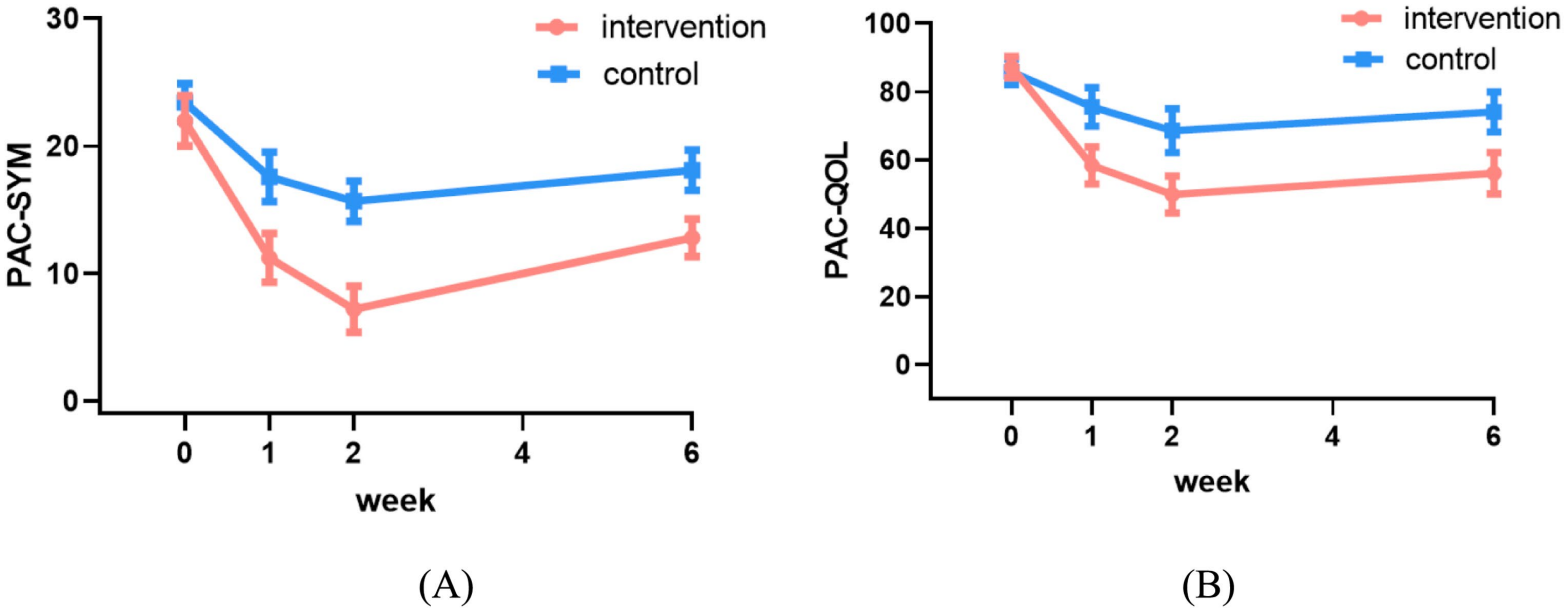
Changes in PAC-SYM **(A)** and PAC-QOL **(B)** scores between the two groups.

Table 6 presents a comparison of PAC-SYM and PAC-QOL scores between the two groups. The significant interactions indicated divergent patterns of improvement between the groups. The intervention group demonstrated a significantly greater reduction in PAC-SYM scores compared to the control group at all follow-up assessments (Week 1: coefficient = −0.36, 95% CI: −0.52 to −0.25; Week 2: coefficient = −0.72, 95% CI: −0.91 to −0.52; Week 6: coefficient = −0.28, 95% CI: −0.39 to −0.18; all *p* < 0.001) (Table 6), indicating superior improvement in constipation symptoms (Figure 5A). Similarly, for quality of life (PAC-QOL), the intervention group showed a significantly greater improvement than the control group at all time points (Week 1: coefficient = −0.27, 95% CI: −0.34 to −0.20; Week 2: coefficient = −0.34, 95% CI: −0.43 to −0.24; Week 6: coefficient = −0.29, 95% CI: −0.38 to −0.21; all *p* < 0.001) (Table 6), demonstrating greater enhancement in constipation-related quality of life (Figure 5B).

**Table 6:** Comparison of PAC-SYM and PAC-QOL scores between the two groups.

Variable	PAC-SYM			p*	PAC-QOL			p*
	B	SE	95% CI		B	SE	95% CI	
Group								
Control	Reference							
Intervention	−0.64	0.53	(−0.17 to 0.04)	0.23	0.02	0.03	(−0.04 to 0.07)	0.34
Time								
Baseline	Reference							
Week1	−0.29	0.04	(−0.36 to −0.22)	<0.001	−0.13	0.02	(−0.16 to −0.09)	<0.001
Week2	−0.40	0.04	(−0.47 to −0.33)	<0.001	−0.22	0.03	(−0.28 to −0.17)	<0.001
Week6	−0.26	0.03	(−0.32 to −0.20)	<0.001	−0.15	0.02	(−0.19 to −0.11)	<0.001
Group × time								
Intervention × week1	−0.36	0.07	(−0.52 to −0.25)	<0.001	−0.27	0.04	(−0.34 to −0.20)	<0.001
Intervention × week2	−0.72	0.10	(−0.91 to −0.52)	<0.001	−0.34	0.05	(−0.43 to −0.24)	<0.001
Intervention × week6	−0.28	0.05	(−0.39 to −0.18)	<0.001	−0.29	0.04	(−0.38 to −0.21)	<0.001

*Calculated using generalized estimating equations.

CSS, Constipation Symptom Scale; CSBMs, complete spontaneous bowel movements; BSFS, Bristol Stool Form Scale; PAC-SYM, Patient Assessment of Constipation-Symptoms; PAC-QOL, Patient Assessment of Constipation Quality of Life; GEE, generalized estimating equations; CI, confidence interval.

### 3.5 Adverse effects

The incidence of adverse effects was significantly lower in the intervention group than in the control group (6.45% vs. 19.35%, *p* = 0.015) (Table 7). Notably, lower cases of nausea or vomiting were observed in the intervention group during the study period.

**Table 7:** Comparison of adverse effects between the two groups.

Group	Nausea	Abdominal bloating	Diarrhea	Overall incidence rate
Intervention group (*n* = 31)	0 (0)	0 (0)	1 (3.22)	1 (3.22)*
Control group (*n* = 31)	1 (3.22)	3 (9.68)	2 (6.45)	6 (19.35)
Z	5.732			
*p*	0.015			

*Compared with the control group, *p* = 0.0045 < 0.05.

## 4 Discussion

The prevalence of FC is high among the older adult, severely affecting their physical and mental well-being (44). FC poses a clinical challenge in the geriatric population, where treatment choices are often complicated by polypharmacy and age-related vulnerabilities (4). Against this therapeutic backdrop, our study investigated an integrative strategy aimed at mitigating the drawbacks of lactulose, specifically its tendency to cause bloating and nausea.

Zhi-Shi plaster, as an external TCM preparation, contains active ingredients that help alleviate gastrointestinal bloating and promote bowel movements. The findings demonstrate that the combination of lactulose and Zhi-Shi plaster acupoint therapy yielded a clinical efficacy rate of 96.77%, which is notably higher than the 74.2% observed in the lactulose-only control group and compares favorably with reported efficacy rates for first-line agents. More importantly, this regimen appears to directly mitigate the limitation of lactulose. The incidence of adverse effects such as nausea and abdominal bloating was Markedly reduced in the intervention group, a marked improvement over the 25.8% rate in the control group. This suggests that the addition of Zhi-Shi plaster may counterbalance the gas-producing fermentation process of lactulose, thereby bringing its tolerability to a level that is competitive with PEG (45). This integrative approach thus presents a valuable alternative for patients who require the efficacy of an osmotic laxative but are intolerant to the side effects of lactulose.

When contextualized with previous research, our results on symptom relief (CSS, PAC-SYM) and quality of life (PAC-QOL) align with studies reporting the benefits of TCM external therapies for functional gastrointestinal disorders (12, 15). However, our study provides a unique contribution by quantifying the synergistic effect of a sustained, non-invasive acupoint therapy when combined with a standard osmotic laxative. The choice of Zhi-Shi plaster, as opposed to other TCM modalities like acupuncture or moxibustion, was strategic. Plaster therapy offers concentrated drug delivery, ease of self-administration, and minimal invasiveness, making it particularly suitable and acceptable for older adult patients with limited mobility (18, 19), thereby supporting adherence and the potential for long-term management.

While our study demonstrated a favorable safety profile for Zhi-Shi plaster acupoint therapy, it is prudent to discuss its foreseeable risks to ensure safe clinical application. The primary risks are associated with its topical and herbal nature. Local skin reactions are the most commonly anticipated adverse effects. These may include contact dermatitis, erythema (redness), pruritus (itching), or localized rash at the application site.

Despite these promising findings, several limitations must be acknowledged. First, participant recruitment was conducted at a single center, which may limit the generalizability of the findings to broader populations. Second, although a placebo-controlled design was implemented, the distinctive herbal aroma of the Zhi-Shi plaster may have compromised blinding among participants in the intervention group. Third, the 2-week intervention period and 4-week follow-up may be insufficient to evaluate the long-term sustainability of treatment effects. Finally, all outcome assessments were based on patient-reported subjective scales, which are susceptible to expectation bias.

## 5 Conclusion

Our findings indicated that compared to lactulose alone, the combination of Zhi-Shi plaster acupoint therapy and lactulose significantly enhanced clinical efficacy in geriatrics FC patients. This approach not only reduces the adverse reactions of lactulose but also effectively alleviates constipation-related discomfort, improves the quality of life, and helps prevent recurrence. These findings offer new perspectives and approaches for improving comprehensive care for geriatrics FC patients.

## Data Availability

The original contributions presented in the study are included in the article/supplementary material, further inquiries can be directed to the corresponding author.
